# Genome-Wide Association Study Dissects the Genetic Architecture of Maize Husk Tightness

**DOI:** 10.3389/fpls.2020.00861

**Published:** 2020-06-30

**Authors:** Siqi Jiang, Haibo Zhang, Pengzun Ni, Shuai Yu, Haixiao Dong, Ao Zhang, Huiying Cao, Lijun Zhang, Yanye Ruan, Zhenhai Cui

**Affiliations:** ^1^College of Bioscience and Biotechnology, Shenyang Agricultural University, henyang, China; ^2^Shenyang Key Laboratory of Maize Genomic Selection Breeding, Shenyang, China; ^3^College of Plant Sciences, Jilin University, Changchun, China

**Keywords:** maize (*Zea mays*), genome-wide association study, husk tightness, genetic architecture, SNPs

## Abstract

The husk is a leafy outer tissue that encloses a maize ear. Previously, we identified the optimum husk structure by measuring the husk length, husk layer number, husk thickness and husk width. Husk tightness (HTI) is a combined trait based on the above four husk measurements. Unveiling the genetic basis of HTI will aid in guiding the genetic improvement of maize for mechanical harvesting and for protecting the ear from pest damage and pathogen infection. Here, we used a maize associate population of 508 inbred lines with tropical, subtropical and temperate backgrounds to analyze the genetic architecture of HTI. Evaluating the phenotypic diversity in three different environments showed that HTI exhibited broad natural variations and a moderate heritability level of 0.41. A diversity analysis indicated that the inbred lines having a temperate background were more loosely related than those having a tropical or subtropical background. HTI showed significant negative correlations with husk thickness and width, which indicates that thicker and wider husks wrapped the ear tighter than thinner and slimmer husks. Combining husk traits with ∼1.25 million single nucleotide polymorphisms in a genome-wide association study revealed 27 variants that were significantly associated with HTI above the threshold of P < 7.26 × 10^–6^. We found 27 candidate genes for HTI that may participate in (1) husk senescence involving lipid peroxidation (*GRMZM2G017616*) and programmed cell death (*GRMZM2G168898* and *GRMZM2G035045*); (2) husk morphogenesis involving cell division (*GRMZM5G869246*) and cell wall architecture (*GRMZM2G319798*); and (3) cell signal transduction involving protein phosphorylation (*GRMZM2G149277* and *GRMZM2G004207*) and the ABSISIC ACID INSENSITIVE3/VIVIPAROUS1 transcription factor (*GRMZM2G088427*). These results provide useful information for understanding the genetic basis of husk development. Further studies of identified candidate genes will help elucidate the molecular pathways that regulate HTI in maize.

## Introduction

Maize is the number one production crop in China and in the world, it plays a vital role in ensuring global food security (Lawrence et al., 2008; Shaokun et al., 2017). The maize husk is the leaf-like outer covering of the ear. Husk tightness (HTI) is an important husk trait that plays an important role in ear growth. HTI is negatively correlated with the water content of the ear after physiological maturity (Li et al., 2014). Loose husks result in faster cob and ear drying rates compared with normal or tight husk (Hicks et al., 1976). The high grain moisture content in the temperate zone results in difficulties during mechanized harvesting, grain drying, and grain storage. Breeding varieties with rapid physiological maturation rates and low water contents, which are suitable for mechanized harvesting, is a serious goal. However, the husk also protects the ear from pest damage and pathogen infection (Barry et al., 1986; Warfield and Davis, 1996). For instance, compared to loose-husked maize, the tight-husked maize have significantly less aflatoxin contamination which is caused by the fungal pathogen *Aspergillus flavus* in maize kernels at pre-harvest stage (Barry et al., 1986; Mcmillian et al., 1987; Manoza et al., 2017). In particular, in subtropical and tropical areas, ear rot is a serious issue during maize ear development (Renfro and Ullstrup, 1976; Afolabi et al., 2007). Tight-husked maize is more resistant to ear rot than loose-husked maize. Therefore, dissecting the genetic basis of HTI would aid the genetic improvement of maize for mechanical harvesting and for protecting the ear from pest damage and pathogen infection.

HTI is a comprehensive trait that may be affected by other physical measurements, such as husk layer number (HN), husk weight, husk length (HL), husk width (HW), and husk thickness (HT). Husk-related traits are the most direct factors affecting the rate of grain dehydration after physiological maturity (Hicks et al., 1976; Cavalieri and Smith, 1985; Li et al., 2014). Several morphological husk traits are negatively correlated with the grain dehydration rate, including HTI (Hicks et al., 1976), husk dry weight (Cross, 1985), HN (Cross, 1985), HW, HL (Crane et al., 1959), HT (Zuber et al., 1950) and husk area. Physiologically, husk moisture is positively correlated with grain moisture (Cavalieri and Smith, 1985), and the husk dehydration rate is positively correlated with the grain dehydration rate (Li et al., 2014). There is also a correlation between the date of husk death and the grain dehydration rate (Cavalieri and Smith, 1985). However, so far, there have been no reports on the correlations between the HTI and other husk-related traits.

Husk traits show broad variations that are subject to genetic regulation and have dissimilar characteristics depending on the genetic background (Zhou et al., 2016). The HN, husk fresh weight, husk dry weight, HL, HW, HT and the total area of the husk are mainly controlled by additive genetic effects (Ping et al., 2000; Dan et al., 2001; Gui-Hua et al., 2015). Most husk studies have been focused at the phenotypic and physiological levels, with limited analyses on the genetic level. Genome-wide association studies (GWASs) are based on linkage disequilibrium and use populations with a wide range of natural variations and a large number of single nucleotide polymorphisms (SNPs) to identify target traits, providing the opportunity to methodically analyze the genetic architecture of complex traits in maize (Flint-Garcia et al., 2003; Aranzana et al., 2005; Li Q. et al., 2012). Compared with conventional QTL mapping, GWAS avoids the difficulty of screening large biparental mapping populations (Su et al., 2016) and has an advantage on identifying the genetic basis of quantitative traits (Schon et al., 2004; Li et al., 2017). Zhou et al. (2016) evaluated 253 maize inbred lines in three environments to detect SNPs for HN and weight. Cui et al. (2016, 2018) used a maize association panel of 508 inbred lines with tropical, subtropical, and temperate backgrounds to decipher the genetic architecture attributed HL, HN, HW, and HT. However, studies on the genetic basis of HTI have not been reported.

In this study, 508 inbred lines of a maize association population which is genotyped with 1.25 million SNPs was used to conduct a GWAS of the HTI in three environments, with the aim of interpreting the phenotypic diversity and genetic basis of HTI. We also analyzed the correlations between HTI and other agronomic traits, including the four husk traits HL, HN, HW, and HT. A series of candidate genes that are associated with husk growth were identified, providing a useful resource for further functional studies.

## Materials and Methods

### Association Mapping Panel

The population used for the GWAS contained 508 diverse maize inbred lines. Among them, 60 inbred lines were from the Germplasm Enhancement of Maize, 223 were from the International Maize and Wheat Improvement Center and 225 were germplasm resources from China. Most of the inbred lines from the International Maize and Wheat Improvement Center are tropical and subtropical, while inbred lines from the United States and China are mostly temperate. Previous research on the kinship of the 508 maize inbred lines was conducted using *k* (model-based subgroups), and the related groups were divided into four subgroups: 27, 70, 196, and 215 inbred lines were placed in the stiff stalk (SS), 70 inbred lines in the non-stiff stalk (NSS), tropical-subtropical (TST), and admixed (MIXED) subgroups, respectively (Yang et al., 2011). Details on the 508 inbred lines including population structure, population divergence, genetic diversity, are available in previously published studies (Yang et al., 2011).

### Field Experiments

All of the 508 inbred lines of the association panel were planted in three environments in China: at Sanya City (SY), Hainan Province in 2015 (15SY) and 2016 (16SY) located in southern China (108°39′E, 18°24′N), and at Fushun City (FS), Liaoning Province in 2016 (16FS) located in northeastern China (121°74′E, 42°14′N). All the lines were planted using a randomized complete block design with two replicates. Each line was planted in single row per plot, 2-m long and 0.6-m wide, with a 0.4-m aisle in the middle.

At 50 d after pollination, the HTIs in the middle of the husks of six plants with similar growth levels were measured using a soft meter ruler. The HTIs were calculated using the following formula:

HTI=[(loosehuskperimeter-tighthuskperimeter)/

loosehuskperimeter]×100%,

where loose husk perimeter refers to the perimeter of the maize ear wrapped with husk in a natural state measured by a soft meter ruler; tight husk perimeter refers to the perimeter of the maize ear wrapped with husk that is tightly attached to the ear after tightening the meter ruler to the smallest possible perimeter. The greater the HTI value, the looser the husk. Conversely, the smaller the HTI value, the tighter the husk.

### Statistical Analysis of Phenotypes

The phenotypic variation of HTI was analyzed using R software 3.5.3. An ANOVA of all the HTI values in the association panel was performed using the aov function in R. The variance analysis of all the HTI data was calculated using the following mixed linear model:*y_ijk_* = μ + *e_i_* + *r(e)_ij_* + *f_k_* + f*e _ik_ + ε*_ilk_*, where μ represents the grand mean of HTI, *e*_i_ represents the environmental effect of the *i*th environment, *r(e)_ij_* represents the effect of the *j*th replication within in the *i*th environment, *f*_k_ represents the genotypic effect of the *k*th line, f*e_ik_ represents the interaction effect between genetic and environmental effects, and ε*_ilk_* represents the residual error. The best unbiased linear predictive value (BLUP) analysis of HTI was also calculated using a mixed linear model, with the average plus the estimated value resulting in the final BLUP value.

The broad-sense heritability is calculated as follows: *h^2^* = σ*_g_^2^*/(σ*_g_^2^* + σ*_ge_^2^*/*e* + σ*_e_^2^*/*re*), where σ*_g_^2^* is the genetic variance, σ*_ge_^2^* is the interaction of genotype with environment, σ*_e_^2^* is the residual error, *e* and *r* represent the number of environments and replications in each environment (Knapp et al., 1985). The estimates for σ*_g_*^2^, σ*_ge_*^2^, σ*_e_*^2^ were obtained by the PROC MIXED procedure in SAS software (Release 9.1.3; SAS Institute, Cary, NC, United States).

### Genome-Wide Association Mapping and Phenotypic Variance Contributions of Significant Loci

We saved the original numbers and BLUP values in the Tab separator format and performed the GWAS analysis using a published genotype with 1,253,814 SNP markers (minor allelic frequency > 5%) (Haijun et al., 2017). The genotype can be downloaded from www.maizego.org/Resources, and it was combined with the 50K SNP array, 600K SNP array, RNA-Seq, and genotyping by sequencing into a whole genetic map (Haijun et al., 2017). The fixed and random model circulating probability unification (FarmCPU) computational method was used to perform the association analysis for HTI, and it separated the multiple loci linear mixed model into fixed and random effect models to reduce false negatives that might result from a confounding population structure, kinship and SNPs (Cui et al., 2016; Liu et al., 2016). The linkage disequilibrium (LD) of the entire panel and four subgroups was analyzed using PopLDdecay software (Chi et al., 2018). The principal component analysis (PCA) of 508 maize inbred lines was conducted using GCTA software (v 1.26.0).

Because of the LD between SNPs in the GWAS, the effective number of independent markers for the adjustment of multiple markers was used to obtain the *P-*value thresholds (Li M. X. et al., 2012). In total, 137,771 markers in approximate linkage equilibrium were found using PLINK (Purcell et al., 2007), and the threshold of LD coefficient, *R*^2^ >0.2, which was discussed and used by Mao et al. (2015). Then, we used the uniform Bonferroni-corrected threshold of α = 1 for the mixed linear model’s significance cutoff as reported in previous studies (Hui et al., 2013; Ning et al., 2014; Mao et al., 2015). Therefore, the suggested *P-*value was computed with 1/n (*n* = 137,771), and we obtained a *P-*value of 7.26 × 10^–6^ for FarmCPU (Liu et al., 2016).

The contribution of SNP to phenotypic variance was calculated using R function “anova(),” with taking the population structure into account. The contribution of each significant SNP was calculated as equation (1), and that of all significant SNPs together was calculated as equation (2).

(1) $$Y=X_{i}\,\alpha_{i}+P\,\beta +\varepsilon$$

(2) Y=X⁢α+P⁢β+ε

where Y is a vector (n*1) for phenotype; *X*_i_ is a vector (n*1) representing the genotype of the *i*th significant SNP; *α_i_* is a number representing the marker effect for *X*_i_. *X* is a matrix (n*p) representing the genotype of all significant SNPs; α is a vector (p*1) of marker effects for *X*; *P* is a matrix (n*2) indicates the subpopulation structure (NSS, SS, and TST); β is a vector (2*1) for the subpopulation structure effect; ε is a vector (n*1) for the residuals; n is the number of individuals; p is the number of significant SNPs.

### Annotation of Candidate Genes

The most significant SNP was chosen to represent the locus in the same LD block d (*r*^2^ < 0.2). The physical locations of the SNPs were determined in reference to the B73 RefGen_v2^1^. Annotated genes within a 50-kb range upstream and downstream of the significantly associated SNP locus were searched for and identified based on functional domains. If the genes in this segment or their homologs in Arabidopsis were involved in the maize leaf metabolic pathway, the gene was predicted to be a candidate gene. If there were no synthesis and degradation pathways involved in maize leaf composition in the segment, the closest gene from the significant site was determined to be a candidate gene.

### Heat-Map of Candidate Genes

Raw datasets of RNA-Seq from different maize tissues were downloaded from NCBI’s Sequence Read Archive database. The details regarding data sources are described in Supplementary Table S1. RNA-Seq reads were aligned to B73 RefGen_v2 using the TopHat (v2.1.0) pipeline with a built-in Bowtie (v0.12.9) mapping program. Only the unique mapped reads were retained for FPKM determination using Cufflinks (v2.2.1). The values used in the heat-map are the log2 transformed ratios of normalized FPKM counts in husk relative to other tissues. The values greater than +3 or less than −3 were adjusted to 3 or −3, respectively.

## Results

### HTI Diversity and Heritability

**FIGURE 1:**
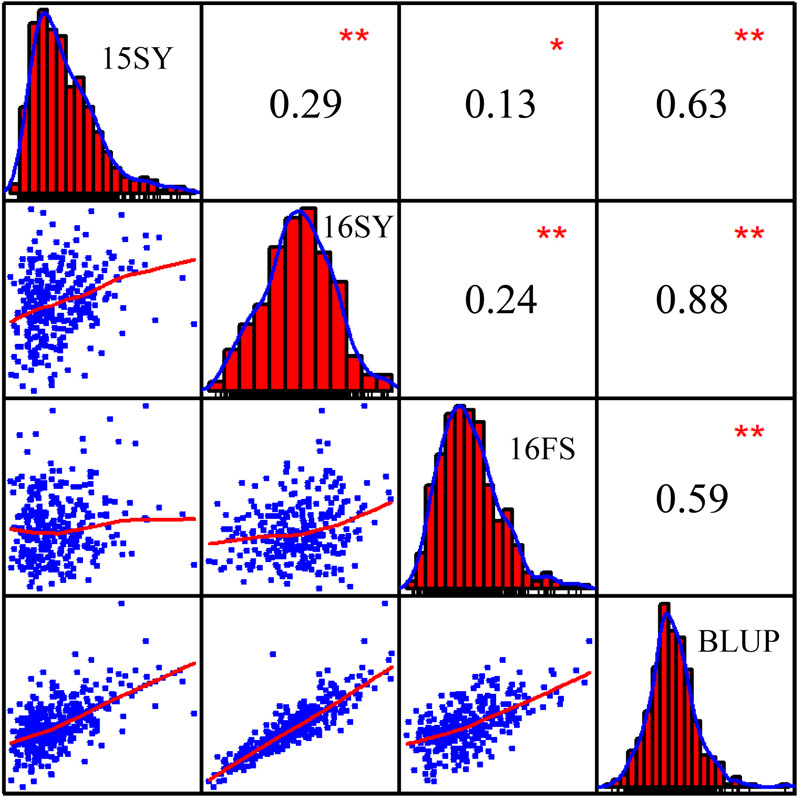
Frequency distributions and correlations for maize husk tightness (HTI) measured in three environments, 15SY (2015 Sanya), 16SY (2016 Sanya), 16FS (2016 Fushun), and BLUP (best unbiased linear predictive value). The plots on the diagonal represent the phenotypic distribution frequency of HTI in 15SY, 16SY, 16FS, and BLUP. The values above the diagonal line are the Pearson’s correlation coefficients between HTIs measured in every two environments. The values below the diagonal line are scatter plots for HTIs measured in every two environments. *Represents a significant difference at the 0.05 level; **Represents a significant difference at the 0.01 level.

The phenotypic data for the 508 lines of the association panel in Hainan and Liaoning Provinces in 2015 and 2016 were measured independently, and the BLUP value was calculated using the phenotypic values of the three environmental field trials as random effects (Supplementary Table S2). The HTIs of 15SY, 16SY, 16FS, and BLUP showed a normal distribution, and there were significant positive correlations among them (*P* < 0.05; Figure 1). The mean heritability of HTI across three environments was estimated as 41% (Table 1).

**TABLE 1:** Variance composition and broad-sense heritability of HTI trait in the maize association population in three environments (15SY, 16SY, and 16FS).

**Source of variation^a^**	**Mean square**	**Significance^b^**	**H^2c^**
Environment (E)	16170.31	<0.01**	0.41
Genotype (G)	27.25	<0.01**	
G × E	13.73	<0.01**	

^a^G × E indicates the interaction between G and E. **Significant at P ≤ 0.01.^c^*Family mean-based broad-sense heritability.*

### Population Structure, Genetic Diversity, and Linkage Disequilibrium (LD)

**FIGURE 2:**
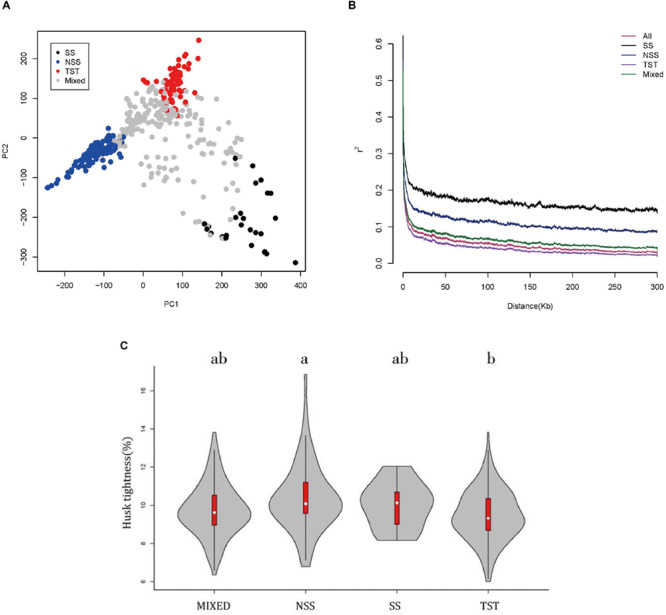
The PCA, linkage disequilibrium and boxplot of maize husk tightness (HTI) distributions in subgroups. **(A)** PCA plot for 508 maize inbred lines. SS green, NSS blue, TST red, Mixed pink. **(B)** Linkage disequilibrium among the entire panel (508lines), SS, NSS, TST, and Mixed subgroups. **(C)** Boxplot of maize husk tightness (HTI) distributions in subgroups. An analysis of variance was used to examine the differences in traits among subgroups. The numbers of inbred lines included in each subpopulation are 215, 70, 27, and 196 for Mixed, NSS, SS and TST, respectively.

The population structure of the association panel used in this study has been fully analyzed by Yang et al. (2011) with 926 SNPs. The association panel was divided into four subgroups, SS, NSS, TST, and Mixed (Yang et al., 2011). The SS and NSS subgroups belonged to the temperate kinship, the TST subgroup belonged to the tropical and subtropical kinship, and the MIXED subgroup contained the remaining non-classified inbred lines (Yang et al., 2011). In this study, an enlarged genotype with 1.25M SNPs was used to perform PCA analysis of the association panel (Figure 2A). The result showed that the panel was divided in to four subgroups, which was consistent with the previous study (Yang et al., 2011). LD across 1.25M SNPs was investigated among the entire panel, SS, NSS, TST, and Mixed subgroups (Figure 2B). A rapid LD decay pattern in the entire panel was observed. And compared with the TST subgroup, LD decayed relatively slow in NSS and SS subgroups. It can be explained by the fact that tropical maize lines have undergone more intense recombination and contain more rare alleles than temperate maize lines (Lu et al., 2011), since the SS and NSS subgroups belonged to the temperate kinship and the TST subgroup belonged to the tropical and subtropical kinship. The contribution of population structure on the HTI phenotype was distinguished by comparisons of changes in the subgroups’ HTIs as visualized in a boxplot (Figure 2C). The HTI of the NSS subgroup was significantly greater than that of the TST subgroup (Figure 2C). In summary, HTI exhibited extensive variation owing to genetic background and differences in population structure.

### Correlations of HTI With Other Plant Developmental Processes

To investigate the correlations between HTI and other agronomic traits, Pearson’s correlation coefficients were calculated after comparing HTI with 21 agronomic traits that had been previously measured in the same association panel, including four husk-related traits, HL, HN, HW and HT, seven morphological traits, plant height, ear height (EH), ear leaf width, ear leaf length (ELL), tassel maximum axis length, tassel branch number, and leaf number above ear, seven yield traits, ear length, ear diameter (ED), cob diameter (CD), kernel number per row, cob grain weight, cob weight, kernel width, and three maturity traits, days to anthesis (DTA), days to silking and days to heading (DTH) (Ning et al., 2014).

**FIGURE 3:**
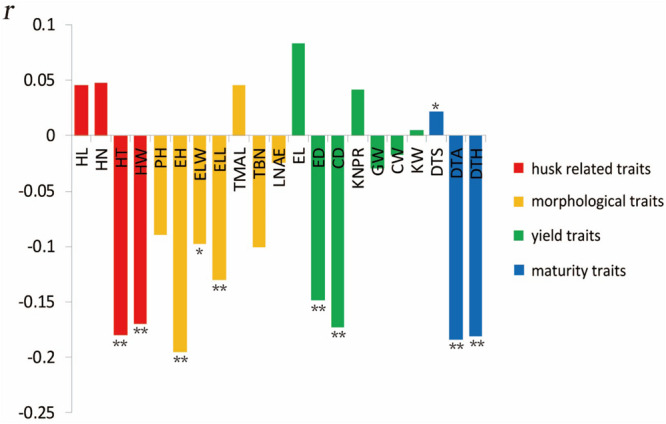
Correlation coefficients of maize husk tightness (HTI) with other husk-related traits based on BLUP values. *Significant at *P* ≤ 0.05; **significant at *P* ≤ 0.01.

The most significant negative correlations were present between HTI and EH (Figure 3). The only significantly positive correlation was observed between HTI and days to silking. Among husk traits, HT and HW exhibited significant negative correlations with HTI, which indicated that a longer or thicker husk contributes to a tighter husk. Among morphological traits, EH, ELL, and ear leaf width showed significantly negative correlations with HTI. Among yield traits, ED and CD displayed significant negative correlation with HTI. Moreover, all three maturity traits exhibited remarkable negative correlations with HTI.

### Genome-Wide Association Analysis

**FIGURE 4:**
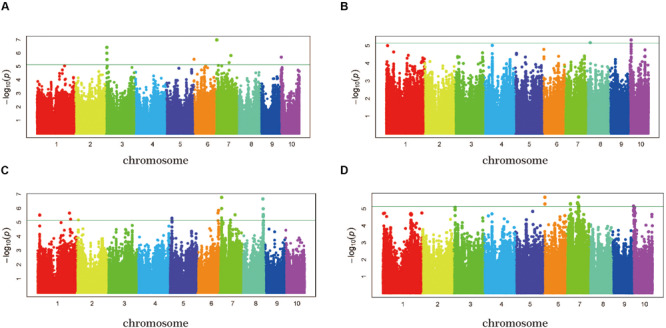
GWAS-derived Manhattan plots showing significant *P*-values associated with husk tightness using FarmCPU and association mapping results, genomic locations, and allele effects of significant SNPs located near representative genes for husk tightness. Each dot represents a SNP. The horizontal dashed blue line represents the Bonferroni-corrected significant threshold of 7.26 × 10^– 6^. **(A)** 15SY; **(B)** 16SY; **(C)** 16FS; **(D)** BLUP.

To reduce the impact of environmental variability, phenotypic BLUP values across three environments (15SY, 16SY, and 16FS) were also used for association studies. The GWAS analysis was performed using 1,253,814 SNP markers (minor allelic frequency > 0.05), with a threshold of *P* < 7.26 × 10^–6^ (Figures 4A–D). When using the FarmCPU for the GWAS analysis, there were seven independent significant SNPs in chr3, -6, -7, and -10 above the threshold in 15SY, accounting for 2.2–8.0% of phenotypic variation, respectively, and 23.7% of phenotypic variation in total (Figure 4A and Table 2). There were two independent significant SNPs in chr8 and chr10 above the threshold in 16SY, accounting for 1.4 and 3.4% of phenotypic variation, respectively, and 4.8% of phenotypic variation in total (Figure 4B and Table 2). There were 13 independent significant SNPs in chr1, 2, and 5–8, above the threshold in 16FS, accounting for 0.3–14.0% of phenotypic variation, respectively, and 48.9% of phenotypic variation in total (Figure 4C and Table 2). There were five SNPs in chr6, 7, and 10 above the threshold in the BLUP analysis, accounting for 3.9–7.6% of phenotypic variation, respectively, and 24.1% of phenotypic variation in total (Figure 4D and Table 2). It was worth noting that the two independent significant SNPs above the threshold in 16SY explained only 4.8% of the phenotypic variation in total. It may be due to the relatively large *P*-value of the two independent significant SNPs in 16SY (Figure 4B and Table 2).

**TABLE 2:** SNP chromosomal positions and candidate genes significantly associated with three maize husk traits identified by GWAS using the FarmCPU method.

**Environment**	**SNP**	**Chr**	**Position (bp)**	**Allele^a^**	***P*-value**	***R*^2^ (%)^b^**	**Gene**	**Gene interval (bp)**	**Annotation**	**Pathway**
15SY	chr3.S_9862250	3	9862250	G/A	3.74E-07	4.3	*GRMZM2G305900*	9852648.9854602	HXXXD-type acyl-transferase family protein	Metabolic
15SY	chr3.S_12295391	3	12295391	G/T	2.99E-06	4.4	*GRMZM2G066413*	12289349.12292072	Phosphoenolpyruvate/phosphate translocator 3, chloroplastic	Cellular transport
15SY	chr6.S_2245399	6	2245399	A/G	2.92E-06	2.2	*GRMZM2G118378*	2241471.2243599	Glycolipid transfer protein 2	Cellular transport
15SY	chr7.S_10969686	7	10969686	C/T	1.06E-07	8.0	*GRMZM2G115357*	10970395.10971721	iaa32 – Aux/IAA-transcription factor 32	Gene expression regulation
15SY	chr7.S_107782578	7	107782578	A/T	5.18E-06	7.5	*GRMZM2G319798*	107780060.107782693	Probable xyloglucan endotransglucosylase/hydrolase protein 28	Metabolic
15SY	chr7.S_121815983	7	121815983	C/G	1.54E-06	6.7	*GRMZM2G004207*	121815664.121817633	Serine/threonine-protein kinase	Signal transduction
15SY	chr10.S_7440298	10	7440298	G/T	2.03E-06	4.2	*GRMZM2G156506*	7465830.7467824	Unknown	Unknown
Total^c^						23.7				
16FS	chr1.S_16579944	1	16579944	C/T	3.03E-06	10.7	*GRMZM2G017616*	16574646.16581385	lox9 – lipoxygenase9	Metabolic
16FS	chr1.S_245869612	1	245869612	G/A	2.22E-06	7.8	*GRMZM2G013832*	245885894.245886606	Unknown	Unknown
16FS	chr1.S_254468476	1	254468476	A/G	6.08E-06	5.9	*GRMZM2G088427*	254427709.254429811	abi30 – ABI3-VP1-transcription factor 30	Gene expression regulation
16FS	chr2.S_12258281	2	12258281	T/G	6.71E-06	6.8	*GRMZM2G074743*	12267202.12268962	aox3 – alternative oxidase3	Metabolic
16FS	chr5.S_22604023	5	22604023	C/G	5.05E-06	0.3	*GRMZM5G869246*	22555032.22556880	Kinesin-like protein KIN-7K, chloroplastic	Cell division
16FS	chr6.S_159150793	6	159150793	G/C	2.18E-06	7.1	*GRMZM2G168898*	159147009.159148433	Hemoglobin 2	Programmed cell death
16FS	chr6.S_163988321	6	163988321	G/A	1.45E-06	6.5	*GRMZM2G451224*	163973641.163974980	psah1 – photosystem I H subunit1	Photosynthesis
16FS	chr7.S_18428776	7	18428776	G/A	1.71E-07	14.0	*GRMZM2G432631*	18416829.18421492	Serine carboxypeptidase-like 51	Metabolic
16FS	chr7.S_85722926	7	85722926	C/T	6.8E-06	5.4	*GRMZM5G818812*	85764523.85765810	Not found	Unknown
16FS	chr7.S_122072472	7	122072472	C/T	2.95E-06	10.9	*GRMZM2G075348*	122114277.122129927	Unknown	Unknown
16FS	chr8.S_159951554	8	159951554	A/G	2.18E-07	4.7	*GRMZM2G302074*	159908755.159910600	Cytochrome P450 89A2-like	Metabolic
16FS	chr8.S_162182867	8	162182867	C/T	1.1E-06	12.3	*GRMZM2G082390*	162171809.162175128	sumo1b – small ubiquitin-related modifier1b	Metabolic
16FS	chr8.S_163509315	8	163509315	A/G	3.45E-06	4.1	*GRMZM2G035045*	163455713.163457350	KDEL-tailed cysteine endopeptidase CEP1	Metabolic
Total^c^						48.9				
16SY	chr8.S_25010301	8	25010301	C/G	6.77E-06	1.4	*GRMZM2G046306*	24983726.24985596	GDSL esterase/lipase At1g28590	Metabolic
	chr10.S_12751979	10	12751979	C/T	4.09E-06	3.4	*GRMZM2G063394*	12744140.12754513	U-box domain-containing protein 43	Metabolic
Total^c^						4.8				
Blup	chr6.S_7038488	6	7038488	C/G	2.07E-06	7.5	*GRMZM2G127294*	7051859.7059857	Lysine histidine transporter 2	Cellular transport
Blup	chr7.S_32631016	7	32631016	G/A	4.97E-06	7.2	*GRMZM2G109743*	32624687.32633165	Dehydrogenase/reductase SDR family member 2	Metabolic
Blup	chr7.S_86923418	7	86923418	A/G	4.37E-06	6.3	GRMZM2G149277	86875469.86876591	Putative leucine-rich repeat receptor-like protein kinase family protein	Signal transduction
Blup	chr7.S_94663353	7	94663353	T/G	2E-06	7.6	GRMZM2G027041	94644611.94646428	Putative cytochrome P450 superfamily protein	Metabolic
Blup	chr10.S_4873724	10	4873724	T/G	6.94E-06	3.9	*GRMZM2G014282*	4896097.4911690	ABC transporter G family member 41	Cellular transport
Total^c^						24.1				

**^*a*^Major/minor allele, underlined bases indicate the favorable alleles. ^*b*^Percentage of phenotypic variation explained by the additive effect of the single significant SNP. ^*c*^Total percentage of phenotypic variation explained by all the significant SNPs.**

### Expression Pattern of Candidate Gene in Different Maize Tissues

**FIGURE 5:**
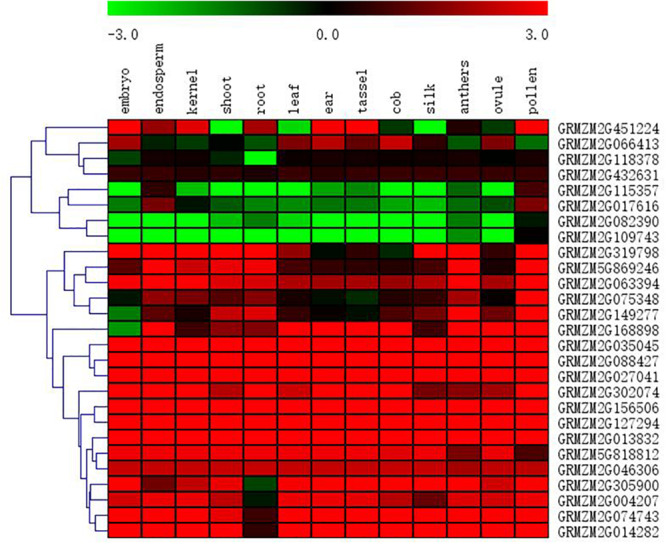
Heat-map of tissue-specific expression patterns of candidate genes identified by GWAS. The values used in the figure are the log2 transformed ratios of normalized PRKM counts in husk tissues to those in other tissues as shown at the bottom of each column. Columns and rows are ordered according to similarity (hierarchical cluster analysis at the top and left). The red, black, and green represent higher, similar, and lower in husk compared with in other tissues, respectively, for a particular gene.

All 27 SNPs significantly associated with HTI were identified by FarmCPU, and 27 candidate genes that were significantly associated with HTI were obtained (Table 2). The 27 candidate genes were divided into eight functional types, metabolic, cellular transport, gene expression regulation, signal transduction, photosynthesis, programmed cell death, cell division and unknown function (Table 2). To determine whether the genes denoted by significant SNPs were specifically expressed in husk tissues, an *in silico* expression pattern was compiled using the published RNA-Seq datasets from 14 different organs/tissues, including husk tissues (Figure 5). In total, 14 candidate genes, *GRMZM2G168898*, *GRMZM2G302074*, *GRMZM2G156506*, *GRMZM2G127294*, *GRMZM2G013832*, *GRMZM2G818812*, *GRMZM2G046306*, *GRMZM2G004207*, *GRMZM2G305900*, *GRMZM2G014282*, *GRMZM2G074743*, *GRMZM2G035045*, *GRMZM2G088427*, and *GRMZM2G027041* had higher expression tendencies in husk relative to other tissues. There were four candidate genes, *GRMZM2G082390*, *GRMZM2G109743*, *GRMZM2G115357*, and GRMZM2G017616, that showed lower expression tendencies in husk relative to other tissues. The high and low expression levels of these candidate genes in husk further suggested their relevance in HTI.

## Discussion

### Genetic Basis of HTI

The heritability of husk related traits in maize such as husk dry weight, husk width, husk thickness, etc. have been reported, and the heritability range is between 0.36 and 0.89 (Dan et al., 2001; Cui et al., 2016; Zhou et al., 2016). In this study, HTI showed a wide range of phenotypic variation and followed a normal distribution (Supplementary Table S2 and Figure 1). A genetic analysis showed that the heritability of HTI was moderate (*h^2^* = 0.41) (Table 1), which indicated that this trait was heritable and is suitable for genome-wide association analysis (Phuthaworn et al., 2016). The Genotype effects for HTI across three environments were significant, indicating the involvement of gene action in the control of HTI (Table 1). The Environment × Genotype interaction effects were also significant for HTI suggesting that the degree of transmission of HTI in maize differed from one location to the other (Table 1). In other words, the genes effects involved in the inheritance of HTI were differential to the environments. Different lines could therefore be selected for specific environment to improve the HTI in a breeding project.

Maize originated in the tropics, and was then domesticated in subtropical and temperate regions. Therefore, the morphological structure of maize is strongly influenced by the population structure (Camus-Kulandaivelu et al., 2006). To investigate the effect of population structure on HTI, the phenotypic variations of husk HTI were compared between different subpopulations (Figure 2C). Increases in mean and scale for the TST subgroup compared to those for the NSS subgroup were observed, which suggests that maize inbred lines of tropical/subtropical origin tend to have tighter husks. This may result from the high temperatures in tropical areas, which aid in the evaporation of water. To prevent the excessive evaporation of water, natural selection favored the tighter husks. On the contrary, owing to the relatively low temperatures in temperate regions, transpiration is relatively weak, and looser husk are conducive to water evaporation. Therefore, maize originating in temperate regions tends to have looser husks.

### Correlations of HTI With Other Plant Developmental Processes

HTI is closely correlated with the morphogenesis of the husk. There are significant negative correlations between HTI and HT, HTI and HW, which indicates that the thicker or wider husk leads to a tighter husk. The wider husk can increase the husk coverage of ears, with the husk wrapping the ear more tightly. In addition, at early stages of dehydration in maize leaves, the leaf thickness is significantly positively correlated with the leaf water content (Búrquez, 1987; Afzal et al., 2017). Thus, the thicker husk generally has a higher water content, which promotes the husks ability to wrap the ears tighter. This explains the negative correlation between HTI and HT.

HTI is negative correlated with some yield and morphological ear-related traits, including CD, ED, ELL, EH, and tassel branch number, which indicates that well-built ears and vegetative growth contribute to the tighter husk. The taller the plant, the longer the leaves, which allows maize to carry out more photosynthesis, contributing more dry matter to the ears and allowing the ears, as well as cobs, to grow larger. The overall framework between husk and ear deliberately cooperate and the husk phenotypes exhibit substantial positive correlations with ear-related traits (Cui et al., 2016). Therefore, the cooperative growth allows the husks to flourish with the ears.

HTI is negatively correlated with DTA and DTH. Thus, the husks become tighter in tandem with the lateness of the maize breeding period. Generally, when the maize breeding period is later, the dehydration of husks is also delayed. Maize lines with relatively late maturation periods have greater husk moisture contents than those with early maturation periods. The greater husk moisture content allows the husks to wrap the ears tighter. However, DTA and DTH also have positive correlations with the HW and HT, respectively (Cui et al., 2016), which indicates that the husk becomes wider and thicker in tandem with an increase in the length of the maize growing period, causing the husk to wrap the ear tighter.

### Candidate Genes and Pathways Involved in HTI

Husk leaves wrap around maize ears and affect the ear water content through physiological mechanisms, such as senescence (Cavalieri and Smith, 1985; Li et al., 2014) and husk morphogenesis (Zuber et al., 1950; Crane et al., 1959; Hicks et al., 1976; Cross, 1985). However, the genetic basis of HTI is unclear. In this study, we identify 26 genes that are significantly correlated with HTI using the FarmCPU method. A functional annotation revealed that these candidate genes could mainly be placed into a few functional groups, such as metabolic, transcriptional regulation and cellular transport. Here, we will discuss how candidate genes may participate in husk senescence, husk morphogenesis and plant growth through different metabolic pathways.

#### Candidate Genes Involved in Husk Senescence

Leaf senescence is an organ-level programmed death process during plant growth and development (Jiang and Rodermel, 1993). In this study, using GWAS, we identified two HTI trait associated candidate genes that regulated programmed cell death (PCD): *GRMZM2G168898*, encoding hemoglobin2, which regulates the death program through mechanisms that interfere with the cascade of events mediated by NO and Zn^2+^, the mitogen-activated protein kinase cascade, and reactive oxygen species (ROS) accumulation that lead to PCD (Huang et al., 2014) and*GRMZM2G035045*, encoding a KDEL-tailed cysteine endopeptidase, which belongs to a subgroup of the papain-type cysteine endopeptidases expressed in tissues undergoing PCD (Höwing et al., 2017). The KDEL-tailed cysteine endopeptidase accepts a wide variety of amino acids at the active site, including the glycosylated hydroxyprolines of the extensions that form the basic cell-wall scaffold (Timo et al., 2014).

The coordinated degradation of biological macromolecules is a main feature in the process of leaf senescence and requires the participation of various enzymes (Thimann, 1980). *GRMZM2G046306* encodes a GDSL esterase/lipase, which belongs to a subclass of lipolytic enzymes with multifunctional properties, such as broad substrate specificity and regiospecificity (Chepyshko et al., 2012). *GRMZM2G432631* encodes a serine carboxypeptidases-like protease, which belongs to a class of proteases in the α/β hydrolase family. Serine carboxypeptidases-like proteases also participate in the degradation of cellular contents in the PCD process that occurs during plant growth (Mc and Cejudo, 2002). *GRMZM2G082390* encodes a small ubiquitin-related modifier1b, which is a member of a family of ubiquitin-related proteins that has several important physiological functions, including antagonizing ubiquitin-mediated protein degradation (Ruijin et al., 2004).

Lipid peroxidation is another main influencing factor on leaf senescence. The oxidative burst, during which large quantities of ROS, like superoxide, hydrogen peroxide, hydroxyl radicals, peroxy radicals, alkoxy radicals and singlet oxygen, are generated, is one of the earliest responses during the natural course of senescence (Elstner, 1982; Khanna-Chopra, 2012). In fact, reactions involving ROS are inherent features of plant cells and contribute to the process of oxidative deterioration that may lead ultimately to cell death (Kellogg and Fridovich, 1975). *GRMZM2G017616* encodes a lipoxygenase, which causes membrane lipid peroxidation. Because lipid peroxidation produces alkoxy, peroxy radicals, as well as singlet oxygen, these reactions in the membrane are a major source of ROS (Bhattacharjee, 2005). The peroxidation of lipids during plant cell senescence can be triggered either by ROS or lipoxygenase, and the activity level of the latter increases with advancing senescence. Thus, lipoxygenase plays a central role in promoting oxidative injury during senescence because it not only initiates a chain reaction of lipid peroxidation, but it can also form singlet oxygen. *GRMZM2G109743* encodes dehydrogenase/reductase SDR family member 2, belonging to the carbonyl-reducing enzymes, and plays an important role in the phase I metabolism of many endogenous products, including those of reactive lipid peroxidation (Hoffmann and Maser, 2007; Kisiela et al., 2017).

#### Candidate Genes Involved in Husk Morphogenesis

In this study, HTI showed negative correlations with HW, HT, and some other agronomic traits. This indicated that the tightness of the leaves was closely correlated with plant growth and leaf morphogenesis.

Leaf size depends on both cell number and cell size, which are controlled by two growth processes called cell division and cell expansion, respectively. The phytohormone auxin (Aux), which controls cell division, expansion and differentiation, is the core of many aspects of plant growth and development. The Aux/IAA transcription factor is involved in leaf morphogenesis and its down regulation results in enhanced Aux sensitivity (Hua et al., 2005). Kinesin-like protein KIN-7K (chloroplastic), encoded by *GRMZM5G869246*, is a target gene of microRNA 160, which is considered to be candidate growth-regulated microRNA that controls the cell division processes indirectly by repressing target genes in maize leaves (Aydinoglua and Lucas, 2018). *GRMZM2G319798* encodes xyloglucan endotransglucosylase/hydrolase (XTH) protein 28, which is a cell wall-modifying enzyme that specifically uses xyloglucan as a substrate (Saladié et al., 2006). Each gene of the XTH family appears to play a particular role in modulating cell-wall architecture in a temporally and spatially specific manner in Arabidopsis and rice (Ryusuke et al., 2004), which indicates that the specific expression of a *XTH* gene might also modulate the cell-wall architecture of husks in maize.

#### Candidate Genes Involved in Cell Signal Transduction

Protein phosphorylation is a fundamental process of cell signal transduction, in which extracellular signals are amplified and propagated by a cascade of protein phosphorylation and dephosphorylation events (Fiene et al., 2017). *GRMZM2G149277* encodes a leucine-rich repeat receptor-like protein kinase family protein, which is reported to actively function in various physiological processes, such as plant growth and development (Kai et al., 2010; Feng et al., 2014). *GRMZM2G004207* encodes a serine/threonine-protein kinase, which is one of the major protein kinases in plants and is a key element involved in signal transduction in responses to metabolism and biotic and abiotic stresses (Kai et al., 2018). Thus, HTI may respond to the environment through this pathway.

*GRMZM2G088427* encodes ABSISIC ACID INSENSITIVE3/VIVIPAROUS1 (ABI3-VP1)-transcription factor 30, which belongs to the B3 DNA-binding domain superfamily (Feng et al., 2014). Although ABI3/VP1 mainly functions in seed development, ABI3 also has broad functions in vegetative growth, such as plastid development, flowering time, and axillary meristem outgrowth (Fischerova et al., 2008; Sakata et al., 2010). VP1 may mediate an interaction between abscisic acid and Aux signaling that alters gene expression patterns (Suzuki et al., 2001).

### Low Expression of Candidate Genes May Play a Role in Husk Morphogenesis

In this study, there are 14 candidate genes which showed higher expression tendencies in husk relative to other tissues and four candidate genes which showed lower expression tendencies in husk relative to other tissues (Figure 5). It is easy to understand that genes with higher expression levels in husk than in other issues, involving in the formation of husk. But genes with lower expression levels in husk than in other issues may also play a role by negative regulating other proteins or genes. For example, *GRMZM2G082390*, one of the four lower expression tendencies in husk relative to other tissues, encodes SUMO-1b protein which has a function in antagonizing ubiquitin-mediated protein degradation. The low level expression of SUMO-1b protein may attenuate the antagonism of proteolysis to promote protein degradation (Ruijin et al., 2004). *GRMZM2G115357* encode Aux/IAA-transcription factor 32, which showed a tendency of lower expression in husk. It was reported that the Aux/IAA transcription factor is involved in leaf morphogenesis and down regulation of the Aux/IAA transcription factor resulted in enhanced auxin sensitivity (Hua et al., 2005). The lower expression of *GRMZM2G115357* may enhance the sensitivity of auxin, so that regulate the husk morphogenesis.

## Conclusion

This study is the first to reveal genetic architecture and mechanisms controlling natural variation in maize HTI by applying GWAS. HTI trait is moderately inheritable, showing a broad variation in a population containing 508 diverse global inbred lines genotyped using 1,253,814 SNP markers. The GWAS demonstrated that there are a number of genetic loci with small effects on regulating the natural variation in HTI. We found 27 candidate genes of HTI that may participate in husk senescence, husk morphogenesis and cell signal transduction. The candidate genes provide a precious resource for further studies to dissect the molecular network involved in regulating maize HTI. And the identification of SNPs will be helpful in facilitating marker-assisted selection of maize HTI in breeding programs.

## Data Availability Statement

All datasets generated for this study are included in the article/Supplementary Material.

## Author Contributions

YR and ZC conceived and supervised the project. SJ and HZ conducted the experiments. PN, SY, HD, AZ, HC, and LZ performed the bioinformatics and statistical analyses. SJ wrote the manuscript. All authors read and approved the final manuscript.

## Conflict of Interest

The authors declare that the research was conducted in the absence of any commercial or financial relationships that could be construed as a potential conflict of interest.

## Footnotes

1 www.maizesequence.org

## Abbreviations

15SY: 2015 Sanya
16FS: 2016 Fushun
16SY: 2016 Sanya
CD: cob diameter
DTA: days to anthesis
DTH: days to heading
ED: ear diameter
EH: ear height
ELL: ear leaf length
HL: husk length
HN: husk number
HT: husk thickness
HTI: husk tightness
HW: husk width.
