# Supplementary material for: Genome-Wide Association Study Dissects the Genetic Architecture of Maize Husk Tightness
Source: Front Plant Sci. 2020 Jun 30;11:861. doi: 10.3389/fpls.2020.00861 (PMC7338587; doi:10.3389/fpls.2020.00861)
Supplement: Supplementary file 1 [file Data_Sheet_1.docx]

Supplementary table1

RNA-Seq data used for the candidate genes’ expression pattern analyses in different maize tissues

| Tissue Samples | Description |
| --- | --- |
| SRR1168430 | B73 embryo |
| SRR1169636 | B73 endosperm |
| SRR1170941 | B73 kernel |
| SRX012380 | B73 shoot |
| SRX012381 | B73 root |
| SRR504466 | B73 leaf |
| SRR504476 | B73 ear |
| SRR504471 | B73 tassel |
| SRR189763 | B73 cob |
| SRR189772 | B73 silk |
| SRR189769 | B73 anther |
| SRR189770 | B73 ovule |
| SRR189771 | B73 pollen |
| SRS394626 | B73 husk |

Supplementary table2

Phenotypic description of HTI trait in the maize association population

| Site^a^ | Means±SD（%） | Range (%) |
| --- | --- | --- |
|  |  |  |
| 15 SY | 6.91±3.42 | 1.81-20.56 |
| 16 SY | 15.83±2.24 | 10.47-21.73 |
| 16 FS | 6.26±1.53 | 3.31-12.05 |
| BLUP | 9.74±1.42 | 6.00-16.86 |

^a^ 15SY, 2015 Sanya; 16SY, 2016 Sanya;16FS, 2016 Fushun; BLUP, the Best Unbiased Linear Predictive value of 15SY, 16SY and 16FS
